# Prevalence and determinants of obstetric violence during facility-based childbirth among postnatal women in Egypt

**DOI:** 10.1038/s41598-026-64411-0

**Published:** 2026-08-17

**Authors:** Nehal Mohamed Eisa, Sarah Sabry, Yomna Youssef, Mohamed Abdeltawab, Ahmed Essam Abou Warda, Mahmoud M. Samir, Marwa M. Gaballah, Reem S. Mahmoud, Abeer Elsayad Elassi, Mai K. Ammar, Abdelrahman S. H. Refaee

**Affiliations:** 1 Clinical Research Department at Giza Health Affairs Directorate, MOHP, Giza, 12585 Egypt; 2 Department of Pharmacy Practice, Faculty of Pharmacy and Drug Technology, Egyptian Chinese University, Cairo, 11771 Egypt; 3 Department of Clinical Pharmacy, 15 May Hospital, Cairo, 11747 Egypt; 4 Clinical Pharmacy Department, Faculty of Pharmacy, October 6 University, Giza, 12585 Egypt; 5 ICH-CRC, MOHP, Giza, Egypt; 6 Department of Pharmacognosy, Faculty of Pharmacy and Drug Technology, Egyptian Chinese University, Cairo, 11771 Egypt

**Keywords:** Psychology, Health care

## Abstract

Obstetric violence (OV) is a significant public health concern involving disrespectful, abusive, and non-consented practices during maternity care. This cross-sectional study investigated its prevalence and determinants among 235 postnatal women in hospitals under the Giza Health Affairs Directorate, Egypt. Women aged 18 years or older who delivered and were preparing for discharge were enrolled; those with loss of consciousness during hospitalization were excluded. Face-to-face interviews used a structured questionnaire covering sociodemographic data, obstetric history, seven domains of OV, and patient satisfaction. Data were analyzed using descriptive statistics, chi-square tests, and logistic regression (*p* ≤ 0.05). The primary outcome was OV prevalence (any affirmative response to violence-related questions); the secondary outcome was identifying factors associated with OV. Of 235 women, 39.15% experienced OV, mainly prolonged waiting (11.91%) and verbal abuse (9.36%), with rare non-consented interventions. No significant associated factors were found. Satisfaction was high for compassionate care (86.81%) and control (85.53%), while 31.91% were dissatisfied with problem resolution. This study identified a moderate prevalence of OV among women, highlighting the need to strengthen respectful maternity care practices through policy improvements, healthcare provider training, and system-level interventions.

**Supplementary Information:**

The online version contains supplementary material available at 10.1038/s41598-026-64411-0.

## Introduction

Obstetric violence (OV) represents a critical public health issue, encompassing a spectrum of disrespectful, abusive, and non-consented practices during childbirth that compromise the dignity and rights of women. The World Health Organization (WHO) underscores that every woman is entitled to dignified, respectful care throughout pregnancy, childbirth, and the postnatal period, and defines OV as any harmful action directed toward women during these stages^1^.

The manifestations of OV are multifaceted. They span physical aggression such as slapping or forced interventions without anesthesia to psychological abuses, including humiliation, verbal degradation, and a breach of privacy and confidentiality. Additionally, OV may involve non-consensual practices during examinations or interventions, leading to both physical discomfort and lasting psychological trauma^2^. These forms of violence can occur during any phase of maternity care, including pregnancy, labor, and the postnatal period, and are not confined to either public or private healthcare facilities.

Empirical studies from diverse global contexts illustrate the widespread nature of this issue. In Tanzania, for example, over 50% of postnatal women reported experiencing at least one form of OV during facility-based childbirth^3^. Similarly, research conducted in Spain revealed that approximately 38% of women encountered OV during childbirth^4^.

In Ethiopia, a systematic review reported that disrespect and abuse during childbirth ranged from 20% to 79% across different studies^5^. In Kenya, more than 60% of women reported experiencing at least one form of mistreatment during facility-based delivery^6^. Within the Arab region, studies from Lebanon and Jordan have also documented high rates of non-consented care and verbal abuse during childbirth, highlighting that OV is a regional concern rather than isolated to a single country^7^.

In Egypt, despite improvements in maternal health services, OV remains underexplored. Preliminary evidence indicates that up to 77% of women experience physical abuse during childbirth, highlighting the urgent need to address this significant public health concern^8^. These findings suggest that OV is not only a grave violation of women’s rights but also a significant barrier to achieving quality maternal care. The normalization of such practices within healthcare systems further complicates their recognition and redress, potentially deterring women from seeking institutional care in future pregnancies.

In Egypt, maternal healthcare is predominantly physician-led. Recent estimates indicate that physicians attend approximately 88% of births, while midwives and nurses attend only about 3%, and traditional birth attendants (dayat) attend 7.5%^9^. Antenatal care is delivered through a network of primary health units and hospitals, with a recommended minimum of five visits per pregnancy. High-risk pregnancies are referred from primary units to general or university hospitals. Most deliveries (92%) occur in health facilities, with caesarean sections accounting for 52% of facility-based births nationally^10^. The majority of women (94% in our sample) were discharged within three days postnatal, reflecting routine clinical practice.

Given the critical implications for both maternal health outcomes and human rights, it is imperative to comprehensively assess the prevalence and determinants of OV. This study aims to fill the existing knowledge gap by systematically evaluating the experiences of OV among postnatal women in hospitals within the Giza Health Affairs Directorate in Egypt.

## Methodology

### Study design and setting

A cross-sectional study was undertaken between August and October 2024 in hospitals under the Giza Health Affairs Directorate. A convenience sampling technique was employed. All eligible postnatal women attending the participating hospitals during the study period who met the inclusion criteria and provided informed consent were invited to participate until the end of the study period. This design enabled a systematic assessment of OV as experienced by postnatal women at the point of discharge. This study was conducted and reported in accordance with the Strengthening the Reporting of Observational Studies in Epidemiology (STROBE) guidelines for cross-sectional studies. A completed STROBE checklist indicating the location of each reporting item has been provided in Supplementary Material 1-Table S1.

### Ethical considerations

The study protocol received approval from the Research Ethics Committee of the Central Directorate for Research and Health Development (REC No. 13-2023/26). Written informed consent was obtained from all participants after a detailed explanation of the study’s objectives, procedures, and confidentiality measures, in accordance with the Declaration of Helsinki.

### Patient selection and recruitment

Women aged 18 years or older who delivered in the participating hospitals and received a discharge decision were eligible for inclusion. Women who experienced loss of consciousness during their hospital stay were excluded to minimize recall bias and ensure the reliability of self-reported information.

Participants were recruited consecutively at the time of discharge from the participating hospitals. Trained research personnel approached all eligible women, explained the study objectives, and invited them to participate. Data were collected through face-to-face interviews conducted by trained research personnel. All participants self-identified as women. Accordingly, the term “women” is used throughout this manuscript.

### Sample size estimation

No formal a priori sample size calculation was performed. All eligible postnatal women who met the inclusion criteria and consented to participate during the study period (August–October 2024) were consecutively recruited, resulting in a final sample of 235 participants. Although this sample allowed estimation of OV prevalence in the study population, the study may have been underpowered to detect small associations between OV and potential predictor variables.

### Data collection

A structured, pretested questionnaire was developed for the purpose of this study. The questionnaire was divided into four sections: (I) sociodemographic data, (II) obstetric history, (III) OV experiences across the seven domains, and (IV) patient satisfaction. Questionnaire items were adapted from previously published OV studies, particularly the instrument used by Mihret et al. (2019)^11^, and were culturally adapted for use in the Egyptian maternity care context. The OV experiences part of the questionnaire was developed using the Bowser and Hill typology of disrespect and abuse during facility-based childbirth^12^. The OV section comprised 22 items distributed across the seven domains: physical abuse (4 items), non-consented care (5 items), non-confidential care (3 items), non-dignified care (4 items), discrimination (2 items), neglect/abandonment (2 items), and detention (2 items).

Patient satisfaction was assessed using six statements adapted from the validated Arabic Childbirth Care Satisfaction Survey (CCSS)^13^. The selected items represented domains corresponding to those covered in our study, including perceptions of control over care, caring and compassionate behavior of healthcare providers, effectiveness of problem management, timeliness of care, appropriateness of care received, and preference for the same type of care in future pregnancies. The original instrument was culturally validated for Arabic-speaking populations and demonstrated acceptable psychometric properties. Responses were rated on a 7-point Likert scale ranging from 1 (strongly disagree) to 7 (strongly agree). Participants were categorized as “satisfied” if the mean score was ≥ 5 and “not satisfied” if the mean score was ≤ 4. Therefore, no further psychometric testing was conducted in the present study. The operational definitions and categorization of all study variables included in the regression analyses are presented in Supplementary Material 1-Table S2.

Face and content validity were assessed by a panel of three experts in obstetrics and public health who reviewed the questionnaire for relevance, clarity, comprehensiveness, and appropriateness of the items. Revisions were made based on expert feedback to improve wording and contextual suitability. In addition, the questionnaire was pretested prior to the main study to assess item clarity, comprehensibility, and feasibility of administration among the target population.

### Study outcomes

The primary outcome was the prevalence of OV among postnatal women. The secondary outcome involved identifying factors associated with the occurrence of OV. As per the protocol, experiencing any one type of violence (i.e., a yes response to any question in the violence section) was used to define OV.

### Statistical analysis

Data analysis was performed using GraphPad Prism. To calculate the overall magnitude (prevalence) of OV, a binary composite variable was constructed based on the 22 items mapping to the seven behavioral domains. A participant was classified as having experienced OV if she gave an affirmative (‘Yes’) response to any one or more of the 22 indicators. Participants who responded negatively (‘No’) to all 22 items were categorized as not having experienced OV. The overall magnitude was expressed as a percentage (n/Nx100) of the total sample (*N* = 235). Continuous variables were tested for normality using the Shapiro–Wilk and Kolmogorov–Smirnov tests and were summarized as means ± standard deviations or medians with ranges, as appropriate. Categorical variables were presented as frequencies and percentages. Comparisons between women who experienced OV and those who did not were performed using Pearson’s chi-square test for categorical variables, while Fisher’s exact test was used when the assumptions of the chi-square test were not met (i.e., when expected cell counts were less than five). Univariable logistic regression analyses were conducted to assess the association between OV and each independent variable separately. Crude odds ratios (CORs) with 95% confidence intervals (95% CIs) were calculated to estimate the strength of these associations. P-values for the logistic regression analyses were derived from Wald tests. A two-sided *p*-value < 0.05 was considered statistically significant.

## Results

### Socio‑demographic characteristics

A total of 235 women participated in the study. No participants were excluded during the study period based on the exclusion criteria. The largest group of women (53, 22.55%) was between 35 and 40 years of age. Most of the respondents lived in urban areas (97.02%), and 97.45% were married at the time of the study. 31.49% finished secondary school, 87.23% were housewives, and 59.57% came from a low socioeconomic level. 124 respondents had 3–5 children (52.77%), and the majority of the respondents got married at the ages of 16–24 years (80.43%). As for the husbands, 73 of them finished secondary school (31.06%), and 73.19% of the husbands worked at private businesses. Table 1 shows the socio-demographic characteristics of the women.

**Table 1:** Socio-demographic characteristics of the women.

Variable	Frequency (*n* = 235)	Percentage (%)
Age, yrs		
16–19	9	3.83
20–24	51	21.70
25–29	41	17.45
30–34	51	21.70
35–40	53	22.55
41–45	20	8.51
≥ 45	10	4.26
Residence		
Urban area	228	97.02
Rural area	7	2.98
Marital status		
Married	229	97.45
Widow	5	2.13
Divorced	1	0.43
Educational background		
Illiterate	60	25.53
Primary school	61	25.96
Secondary school	74	31.49
Higher education	35	14.89
Postgraduate studies	5	2.13
Occupation		
Housewife	205	87.23
Governmental employee	12	5.11
Private business	18	7.66
Social level		
Very low	4	1.70
Low	140	59.57
Average	89	37.87
High	2	0.85
Children		
0–2	106	45.11
3–5	124	52.77
6–8	5	2.13
Age at marriage, yrs		
< 16	9	3.83
16–24	189	80.43
25–34	35	14.89
35–45	2	0.85

### Obstetrical characteristics of the women

From the total of 235 mothers, most of the women, 207(88.09%), received antenatal care since early pregnancy (first trimester), and 70.21% of the women attended antenatal care visits more than 4 times, and 223 (94.89%) of the women followed up the pregnancy with a physician. Among the overall women who participated in the study, more than half of the women (52.77%) reported 2–3 pregnancies, and 78.72% of the women gave birth more than 1 time. 91.91% of the participating women gave birth in a governmental hospital, and more than half of the women gave birth using the caesarean section technique, followed by 18.72% who gave birth without assistance.139 mothers (59.15%) reported that 3–4 people attended the birth, and 97.87% reported that the primary birth attendant was a physician. The dominant key person who attended the birth was reported to be male (60%), and more than half the mothers gave birth during the day (55.74%). Finally, 94.04% of the participating mothers reported having spent less than 3 days in the hospital after giving birth. Table 2 shows the obstetrical characteristics of the women.

**Table 2:** Obstetrical characteristics of the women.

Types of questions	Answers	Frequency (*n* = 235)	Percentage (%)
1. Did you receive antenatal care starting from the first trimester of pregnancy?	Yes	207	88.09
	No	28	11.91
2. Number of antenatal care visits	4 or less	70	29.79
	More than 4	165	70.21
3. You received pregnancy follow-up from	Physician	223	94.89
	Midwife	4	1.70
	Intern doctor	7	2.98
	The pregnancy was not followed up	1	0.43
4. Number of pregnancies	1	41	17.45
	2–3	124	52.77
	4 or more	70	29.79
5. Number of births	First time	50	21.28
	More than one	185	78.72
6. Current place of birth	Government hospital	216	91.91
	University hospital	19	8.09
7. Mode of delivery	Caesarean section	132	56.17
	Normal vaginal using assistive devices	25	10.64
	Normal vaginal with an episiotomy	34	14.47
	Normal vaginal without assistance	44	18.72
8. Number of people who attended the birth	1–2	46	19.57
	3–4	139	59.15
	5–8	50	21.28
9. Primary birth attendant	Physician	230	97.87
	Midwife	2	0.85
	Intern doctors	3	1.28
10. Sex of the primary birth attendant	Male	141	60.00
	Female	94	40.00
11. Timing of birth	During the day	131	55.74
	During the night	104	44.26
12. The number of days you spent in the hospital	Less than 3	221	94.04
	3 or more	14	5.96

### Obstetric violence data

**Fig. 1:**
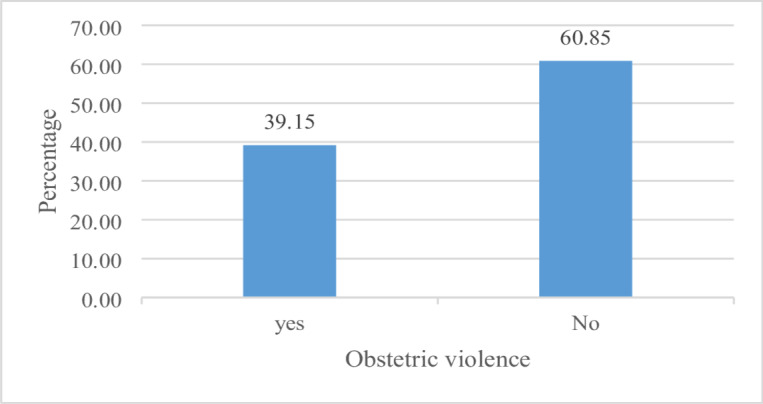
Magnitude of obstetric violence during facility-based childbirth.

From the total of 235 participants, the majority, 143 (60.85%) of women, did not experience OV, while 92 (39.15%) of women experienced at least one form of the different forms of OV (Fig. 1).

Based on the verification criteria for categories of OV, mothers who experienced at least one form of the criteria were counted. The most frequently reported form of OV was prolonged waiting times for medical care, as noted by 28 women (11.91%). The second commonly reported type of OV was experiencing yelling, scolding, or blaming from the birth team (9.36%). This was followed by 8.51% of the mothers reporting being ignored when asking for help during childbirth. The least commonly reported forms of OV were having an episiotomy performed without their consent, having their health information discussed in public places, and having their child detained in the hospital until the money required for the delivery had been paid (1.28% each). However, no participating mothers reported having a hysterectomy or tubal ligation performed or having forceps or a suction device used during the birth without their consent (0.00%). The magnitude of OV during labor and delivery is shown in Table 3.

The least commonly reported domain of OV was non-consented exposure of the woman’s body during childbirth, reported by 7 (2.90%) of mothers. In addition to this, there were only 3 mothers reporting a form of detention in the health care institutions (Table 3). Magnitude of OV during facility-based childbirth is shown in Fig. 1.

**Table 3:** The magnitude of obstetric violence during labor and delivery (*n* = 235).

Obstetric violence	Answer	
	Yes (%)	No (%)
Were you hit, slapped, pinched, or restrained during childbirth?	7 (2.98%)	228 (97.02%)
Was the episiotomy wound sutured without the use of anesthesia?	16 (6.81%)	219 (93.19%)
Was your abdomen pressed hard during childbirth?	15 (6.38%)	220 (93.62%)
Was a vaginal examination performed during childbirth in a violent or unkind manner?	10 (4.26%)	225 (95.74%)
Was a caesarean section performed without your consent?	5 (2.13%)	230 (97.87%)
Was an episiotomy performed without your consent?	3 (1.28%)	232 (98.72%)
Was a hysterectomy performed without your consent?	0 (0.00%)	235 (100%)
Has a tubal ligation been performed without your consent?	0 (0.00%)	235 (100%)
Were forceps or a suction device used during birth without your consent?	0 (0.00%)	235 (100%)
Did the birth take place in a public place without privacy barriers such as curtains?	19 (8.09%)	216 (91.91%)
Did others (other than those in charge of birth) see your body during childbirth?	7 (2.98%)	228 (97.02%)
Did your birth team share information about you and your health with others?	4 (1.70%)	231 (98.30%)
Is your health information discussed in public places?	3 (1.28%)	232 (98.72%)
Did you experience any yelling, scolding, or blaming from the birth team?	22 (9.36%)	213 (90.64%)
Were you treated in a harsh or humiliating manner during childbirth?	12 (5.11%)	223 (94.89%)
Have you been treated in a racist or discriminatory manner for social, educational, financial, religious, or ethnic reasons?	8 (3.40%)	227 (96.60%)
Were your requests for help ignored during childbirth?	20 (8.51%)	215 (91.49%)
Have you noticed that the obstetrician did not attend the delivery process?	5 (2.13%)	230 (97.87%)
Were you treated as a passive participant during the birth process?	8 (3.40%)	227 (96.60%)
Have you waited too long to get the medical care you need?	28 (11.91%)	207 (88.09%)
Were you asked to pay any money for the birth to take place?	14 (5.96%)	221 (94.04%)
Have you or your child been detained in the hospital until the money required for the delivery has been paid?	3 (1.28%)	232 ((98.72%)

### Patient satisfaction

**Fig. 2:**
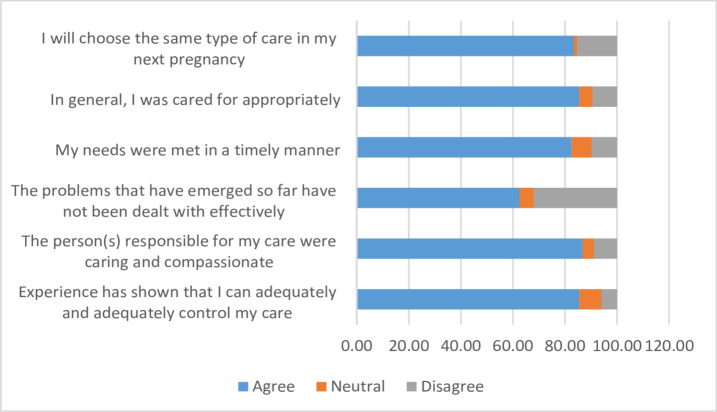
Assessment of patient satisfaction.

The participants’ general satisfaction with the care received during childbirth was assessed. 86.81% of the women generally agreed to having the person responsible for their care be caring and compassionate (*p* < 0.001). Also, the women generally equally agreed to being cared for appropriately and having the ability to adequately control their care (85.53%,

*p* < 0.001). On the other hand, the women generally disagreed with having the problems that have emerged effectively (31.91%, *p* < 0.001) (Table 4; Fig. 2).

**Table 4:** Patient satisfaction assessment.

Statement	Strongly agree *n* (%)	Agree *n* (%)	Somewhat agree *n* (%)	Neutral *n* (%)	Somewhat disagree *n* (%)	Disagree *n* (%)	Strongly disagree *n* (%)	*p*-value
Experience has shown that I can adequately and adequately control my care	64 (27.23)	34 (14.47)	**103 (43.83)**	20 (8.51)	7 (2.98)	3 (1.28)	4 (1.70)	< 0.001
The person(s) responsible for my care were caring and compassionate	**154 (65.53)**	29 (12.34)	21 (8.94)	10 (4.26)	4 (1.70)	5 (2.13)	12 (5.11)	
The problems that have emerged so far have not been dealt with effectively	**71 (30.21)**	38 (16.17)	38 (16.17)	13 (5.53)	8 (3.40)	11 (4.68)	56 (23.83)	
My needs were met in a timely manner	**125 (53.19)**	34 (14.47)	35 (14.89)	18 (7.66)	6 (2.55)	7 (2.98)	10 (4.26)	
In general, I was cared for appropriately	**114 (48.51)**	40 (17.02)	47 (20.00)	12 (5.11)	6 (2.55)	4 (1.70)	12 (5.11)	
I will choose the same type of care in my next pregnancy	**124 (52.77)**	39 (16.60)	33 (14.04)	3 (1.28)	7 (2.98)	9 (3.83)	20 (8.51)	

Pearson’s chi-square test was used for categorical variables, while Fisher’s exact test was applied when expected cell counts were < 5. Statistical significance was defined as *p* < 0.05.

### Factors associated with obstetric violence (univariable logistic regression analysis)

Women aged < 45 years showed lower odds of experiencing OV compared with women aged ≥ 45 years; however, this association was not statistically significant (COR = 0.35, 95% CI: 0.09–1.36, *p* = 0.155). Similarly, place of residence, total number of births, educational status, occupation, staying duration in the hospital, time of birth, sex of the person attending the birth, social level, and current place of giving birth were all not significantly correlated with the probability of OV (Table 5(.

**Table 5:** Univariable logistic regression analysis of factors associated with obstetric violence among women.

Obstetric violence			COR (95%CI)	*p*-value
	Yes	No		
Age of the mother, yrs				
< 45	81 (36.65)	140 (63.35)	0.35 (0.09 to 1.36)	0.155
≥ 45	5 (62.50)	3 (37.50)	1	
Residence				
Urban	89 (39.04)	139 (60.96)	0.85 (0.23 to 3.45)	> 0.999
Rural	3 (42.86)	4 (57.14)	1	
Total births				
One	21 (42.00)	29 (58.00)	1.16 (0.62 to 2.16)	0.744
More than 1	71 (38.38)	114 (61.62)	1	
Educational status				
Illiterate	21 (8.94)	39 (16.60)		0.201
Primary school	23 (9.79)	38 (16.17)		
Secondary school	30 (12.77)	44 (18.72)		
Higher education	18 (7.66)	17 (7.23)		
Postgraduate studies	0 (0.00)	5 (2.13)		
Occupation				
Housewife	80 (4.04)	125 (53.19)		0.983
Governmental employee	5 (2.13)	7 (2.98)		
Private business	7 (2.98)	11 (4.68)		
Staying duration				
Less than 3	88 (39.82)	133 (60.18)	1.66 (0.54 to 4.91)	0.574
3 or more	4 (28.57)	10 (71.43)	1	
Time of birth				
Day	47 (35.88)	84 (64.12)	0.73 (0.44 to 1.26)	0.282
Night	45 (43.27)	59 (56.73)	1	
Sex of attendant				
Female	38 (40.43)	56 (59.57)	1.09 (0.65 to 1.83)	0.786
Male	54 (38.30)	87 (61.70)	1	
Social level				
Very low	1 (0.43)	3 (1.28)		0.372
Low	60 (25.53)	80 (34.04)		
Average	31 (13.19)	58 (24.68)		
High	0 (0.00)	2 (0.85)		
Current place of birth				
Government hospital	82 (37.96)	134 (62.04)	0.5507 (0.2296 to 1.455)	0.228
University hospital	10 (52.63)	9 (47.37)	1	

P-values were derived from Wald tests in univariable logistic regression analyses. P-values were calculated separately for each independent variable by comparing the distribution of women who experienced obstetric violence with those who did not.

## Discussion

This study found that 39.15% of women experienced at least one form of OV, with prolonged waiting (11.91%) and verbal abuse (9.36%) being the most commonly reported forms. No significant associations were identified between OV and the examined sociodemographic or obstetric variables.

Our research contributes valuable data to the limited body of knowledge on OV in Egypt and the broader Eastern Mediterranean Region. It emphasizes that achieving quality maternal care goes beyond ensuring skilled attendance at birth; it requires a comprehensive approach that addresses the multifaceted nature of respectful care.

As for the Socio‑demographic characteristics of our participants, our study found that the majority of respondents (22.55%) were between 35 and 40 years of age. This is higher than what many other studies report. For instance, a study by Benova et al. in Egypt found that only about 10% of births were to women aged 35 or older^14^. Our findings suggest a trend towards later childbearing in our study population, which could have implications for obstetric risks and care needs.

Although Egypt has a substantial rural population, the present study was conducted in hospitals located in Giza, where most participants resided in urban settings. So, the high proportion of urban respondents (97.02%) in our study is not typical of the general Egyptian population. A study by Rashad and Sharaf reported that about 57.00% of Egypt’s population lives in urban areas^15^. Our urban-skewed sample might limit the generalizability of our findings to rural populations and highlight the need for future research focusing on rural maternal experiences.

The high percentage of married respondents (97.45%) is consistent with cultural norms in Egypt but higher than in some other countries. For comparison, a study by Martin et al. in the United States found that about 60.00% of births were to married women^16^. Our findings reflect the strong cultural emphasis on marriage before childbearing in Egypt.

Our finding that 31.49% of mothers finished secondary school is lower than some recent national statistics. The Egypt Demographic and Health Survey 2014 reported that about 43.00% of women had completed secondary education^17^. This difference might reflect regional variations or changes over time in educational attainment.

The percentage of housewives (87.23%) in our study is higher than some other studies in Egypt have found. A study reported that about 70.00% of women in their sample were not employed outside the home^18^. Our higher rate might reflect regional differences or changes in women’s employment patterns.

Our finding that 59.57% of respondents came from low socioeconomic level is important for understanding the social context of our study. This is higher than what some national surveys suggest. For instance, the Egypt Household Income, Expenditure and Consumption Survey 2021 classified about 32.50% of the population as low-income^19^. Our sample might be capturing a more economically vulnerable population.

The finding that 52.77% of respondents had 3–5 children is higher than the national average. The Egypt Demographic and Health Survey 2014 reported a total fertility rate of 3.5 children per woman^9^. Our higher number might reflect regional differences or specific characteristics of our study population.

The majority of our respondents (80.43%) got married between 16 and 24 years. This is consistent with national trends, although the inclusion of marriages under 18 is concerning. A study by Yount et al. in Egypt found that the median age at first marriage for women was 20.8 years, which falls within our most common range^20^.

The educational level and employment status of husbands in our study (31.06% with secondary education, 73.19% in private business) provide important context. These figures are somewhat different from national averages. The Egypt Labor Market Panel Survey found that about 38% of men had secondary education and about 60.00% worked in the private sector. Our sample might reflect specific regional or socioeconomic characteristics^21^.

Collectively, these findings indicate that our study population represents a predominantly socioeconomically disadvantaged group characterized by lower educational attainment, limited female employment, and higher parity compared with national estimates. These characteristics may influence women’s expectations of maternity care, awareness of their healthcare rights, communication with healthcare providers, and willingness to report disrespectful experiences^22^. Consequently, the observed prevalence of OV should be interpreted within the social and economic context of the study population, highlighting the need for interventions that address both healthcare delivery and the broader social determinants of maternal health.

### Obstetrical characteristics of the mother

Our study found that 88.09% of mothers received antenatal care, with 70.21% having more than 4 visits. This is comparable to findings from other middle-income countries. For instance, a study by Benova et al. in Egypt found that 90.00% of women received antenatal care, with 83.00% having 4 or more visits^14^. However, our rates are higher than those reported in some other developing countries. A study by Afulani et al. in Kenya found that only 58.00% of women had 4 or more antenatal visits^23^.

In our study, 94.89% of women received antenatal care from physicians. This is significantly higher than what is reported in many other countries. For example, an integrative review found that in many low- and middle-income countries, a substantial proportion of antenatal care is provided by nurses or midwives^24^. Our findings suggest a highly medicalized model of antenatal care in Egypt.

We found that 78.72% of mothers had given birth more than once. This is higher than what some studies in developed countries report. A study by Martin et al. in the United States found that about 60.00% of births were to women who had previously given birth^25^. Our higher rate might reflect different cultural norms or family planning practices.

Our caesarean section rate of 56.17% is significantly higher than the WHO-recommended rate of 10–15%^26^. However, it’s consistent with trends in Egypt. A study by Elnakib et al. found that the caesarean section rate in Egypt was 52% in 2014^27^. Our rate is much higher than the global averages, as Boerma et al. reported a global average of 21.10%^28^.

This high rate reflects a highly medicalized birth model in Egypt, where physicians attend the vast majority of deliveries^13^. Fear of labor pain among women (56%) and physician attitudes favoring repeat caesareans without trial of labor contribute significantly to this trend^29,30^.

In our study, 97.87% of births were attended by physicians. This is much higher than global averages. Globally, about 81% of births are attended by skilled health personnel, which includes nurses and midwives as well as doctors^16^. Our findings suggest a highly medicalized model of childbirth in Egypt.

It was found that 60% of key birth attendants in our study were male. This contrasts with findings from some other countries where midwifery is more prevalent. For example, a study by Renfrew et al. found that in countries with strong midwifery programs, the majority of birth attendants are female^31^.

94.04% of mothers in our study spent less than 3 days in the hospital after birth. This is consistent with trends in many countries towards shorter postnatal hospital stays. However, it’s shorter than what some studies recommend. A study by Campbell et al. suggested that a minimum of 24 h for vaginal births and 72 h for caesarean sections is beneficial for maternal and neonatal outcomes^32^.

Our finding that 55.74% of births occurred during the day is consistent with patterns observed in other studies. For example, a study by Mathews and Diamond in the US found that births were more likely to occur on weekdays during daytime hours, which they attributed to scheduled inductions and caesarean sections.

Regarding OV, our study shows that 39.15% (95% CI: 32.91%–45.39%) of women experienced at least one form of OV. This prevalence is comparable to what has been reported in some other studies. A systematic review by Bohren et al. found that the prevalence of any mistreatment ranged from 15.00% to 98.00% across studies^33^. Our findings fall within this range. A study in Ethiopia found a prevalence of 62.30%, which is relatively higher than our findings^34^. This emphasizes that regional proximity alone does not necessarily reflect similarities in maternity care practices, healthcare infrastructure, provider–patient relationships, or women’s expectations and perceptions of care.

The most common forms of OV in our study were long waiting times (11.91%), verbal abuse (9.36%), and being ignored when asking for help (8.51%). These findings are consistent with other studies^29^, albeit with some variations in prevalence. For example, a study in Nigeria by Okafor et al. found that the most common forms of disrespect and abuse were non-consented care (54.50%) and detainment in facilities (22.70%), which differ from our primary findings^35^.

Our finding of 9.36% of women experiencing verbal abuse is lower than what some other studies have reported. A study in Tanzania by Sando et al. found that 28.20% of women reported experiencing non-dignified care, including verbal abuse^36^. The lower rate in our study could reflect cultural differences or potentially underreporting.

We found that 8.09% of women reported giving birth without adequate privacy. This is lower than what has been reported in some other studies. For example, a study in Kenya by Abuya et al. found that 58.20% of women reported a lack of privacy^6^. Our lower rate might indicate better infrastructure or policies regarding privacy in Egyptian hospitals.

Our study revealed low rates of non-consented procedures (for example, 2.13% for caesarean sections, 1.28% for episiotomies). These rates are lower than those reported in some other studies. For instance, a study in Ethiopia by Gebremichael et al. found that 9.10% of women reported non-consented care^37^. Our lower rates could indicate better informed consent practices in our study setting.

Non-consented exposure of the woman’s body during childbirth was used as a proxy indicator of sexual violence. We observed a relatively low rate of non-consented exposure during childbirth (2.90%). This finding is consistent with previous studies, which generally report lower frequencies of sexual violence compared with other forms of OV^29^. However, it’s important to note that any occurrence of sexual violence is deeply concerning and requires immediate attention.

Our study revealed a very low frequency of detention-related practices, with only 1.28% of women reporting being detained in healthcare facilities until payment requirements were met. This proportion is lower than the findings reported in a study in Kenya by Warren et al. found that 8.50% of women reported detention in facilities^38^. The difference in this practice highlights differences in healthcare financing systems or policies.

Our study found high levels of patient satisfaction (86.81% agreed that care providers were caring and compassionate) despite the prevalence of OV. This paradox aligns with findings from other studies. For instance, Bohren et al. in their systematic review noted that women often report high levels of satisfaction with maternity care even when they experience disrespect and abuse^39^. This phenomenon could be attributed to low expectations of care or normalization of certain behaviors.

Interestingly, while overall satisfaction was high, 31.91% of participants disagreed that problems were dealt with effectively. This discrepancy is observed in other studies as well. Sando et al. in Tanzania found that women often reported high satisfaction despite experiencing disrespect and abuse, suggesting a complex relationship between perceived quality of care and actual experiences^36^.

The majority of women (83.4%) indicated they would choose the same type of care in their next pregnancy. This high rate of intended return is consistent with findings from other low- and middle-income countries. For instance, Kujawski et al. in Tanzania found that 90.00% of women said they would return to the same facility for future deliveries, despite high rates of disrespect and abuse^40^.

A high proportion of women (82.55%) agreed that their needs were met in a timely manner, which is higher than what some other studies have found. For example, a study by Afulani et al. in Kenya found that only 56.00% of women were satisfied with the timeliness of their care. Our higher satisfaction rates could reflect differences in healthcare system efficiency or in women’s expectations^23^.

The high agreement (86.81%) that care providers were caring and compassionate is encouraging and aligns with the emphasis on respectful maternity care in global health initiatives. However, it contrasts with studies like a study in Ethiopia, which found that only 57.00% of women were satisfied with the empathy shown by their care providers^37^.

The high satisfaction rates in our study, despite the prevalence of OV, may reflect cultural norms and expectations specific to Egypt. Kabakian-Khasholian et al., in their review of women’s experiences of maternity care in the Arab region, noted that women’s perceptions of quality care are heavily influenced by cultural norms and expectations, which may differ from global standards of respectful maternity care^41^.

The paradox of high patient satisfaction despite prevalent OV in our study (86.81% satisfied vs. 39.15% experiencing OV) requires explanation through the lens of Egyptian women’s expectations. Historically, colonial-era health policies marginalized indigenous female practitioners (hakimas and dayas) and privileged male physicians, establishing a paternalistic, male-dominated medical model that persists today^39^. In interwar Egypt, women were publicly blamed for high infant mortality, and motherhood was reconstructed as a national duty through medical and legal discourse^40^. Societally, traditional Egyptian culture has valued women primarily by fertility, with 60.00% illiteracy and only 6.00% workforce participation historically^41^. A pervasive “culture of silence” surrounding reproductive health leads women to normalize conditions such as genital prolapse or reproductive tract infections—and by extension, OV—as “normal” as long as they can bear children and perform daily work^42^.

These low expectations create a “positive disconfirmation” effect, whereby any care that exceeds a very low baseline is rated as satisfactory [43]. In our sample, 58.3% of Egyptian women have been shown to hold negative expectations of nursing support during childbirth^43^. Consequently, traditional satisfaction measures may substantially underestimate quality gaps in respectful maternity care. Our finding of high satisfaction despite prevalent OV reflects not high quality, but rather low expectations shaped by historical marginalization, cultural normalization, and the paternalistic medical system that dominates Egyptian obstetric care.

These findings suggest that interventions aimed at reducing OV should be tailored to the Egyptian sociocultural context, addressing not only healthcare provider practices but also women’s awareness of their rights and expectations regarding respectful maternity care. Such context-specific approaches are likely to be more effective than interventions focusing solely on clinical practice, as they acknowledge the broader social and cultural factors that shape women’s childbirth experiences and reporting of mistreatment.

Our findings contribute to the growing body of literature on OV by providing specific data from the Egyptian context. The prevalent OV (39.15%) challenges the assumption that increased access to skilled birth attendants automatically leads to respectful care. This underscores the need for a more comprehensive theoretical framework that considers not only the presence of medical professionals but also the quality and nature of care provided.

Moving forward, addressing OV should be a priority in Egypt’s efforts to improve maternal health outcomes and experiences. This will require collaborative efforts from policymakers, healthcare providers, and community leaders to create a culture of respectful and dignified maternal care. Based on our findings, we recommend implementing mandatory training programs for all healthcare professionals involved in maternity care, including physicians, nurses, and midwives where applicable, with emphasis on respectful maternity care, effective communication, women’s rights during childbirth, and patient-centered care. In addition, clear protocols and accountability mechanisms should be established to minimize delays and ensure timely care, alongside implementing confidential patient feedback systems that enable anonymous reporting of OV incidents. In addition, awareness campaigns should be implemented to educate women about their rights during childbirth, while hospital policies should be reviewed and revised to ensure alignment with respectful maternity care principles. These measures could significantly reduce the prevalence of OV and improve the overall quality of maternal care in Egypt.

Our cross-sectional study design allowed for a comprehensive snapshot of OV prevalence, but it’s important to acknowledge its limitations. The use of face-to-face interviews may have influenced women’s responses, potentially leading to underreporting due to recent experiences, fear of repercussions, or cultural norms that normalize reproductive health adversity. Additionally, conducting interviews at the time of hospital discharge may have introduced social desirability bias, as women might have been reluctant to report negative experiences while still under the care of the same healthcare providers. Another limitation of this study is that multivariable logistic regression analysis was not performed to adjust for potential confounding factors. Therefore, the reported associations are based on univariable analyses and should be interpreted with caution, as independent predictors of OV could not be identified. However, the interview format also allowed clarification of questions and improved participant understanding. Future research should prioritize including rural populations to provide a more comprehensive picture of OV across diverse geographic settings in Egypt. Future studies might consider a mixed-methods approach, incorporating both quantitative surveys and qualitative interviews to provide a more nuanced understanding of women’s experiences.

In addition, no formal a priori sample size or power calculation was performed. Therefore, the study may have been underpowered to detect statistically significant associations for some variables, and the findings should be interpreted accordingly.

While our study provides valuable insights, it is limited by its focus on urban areas and government hospitals. Future research should expand to rural settings and private healthcare facilities to provide a more comprehensive picture of OV in Egypt. Additionally, longitudinal studies could help understand how women’s perceptions of their birth experiences change over time, addressing the potential underreporting immediately post-birth noted in previous research. Investigating the perspectives of healthcare providers could also offer a more holistic understanding of the factors contributing to OV. Additionally, this study did not assess long-term psychological or physical effects of OV, as it was cross-sectional with no follow-up.

## Conclusion

This study provides crucial insights into the prevalence of OV and its associated factors among postnatal women in Giza, Egypt. Our findings reveal a moderate prevalence of OV, with 39.15% of women experiencing at least one form of OV during childbirth. The most common forms of OV were excessive waiting times, verbal abuse, and being ignored when requesting assistance. These results highlight significant challenges in providing respectful maternity care within the Egyptian healthcare system.

Interestingly, despite the moderate prevalence of OV, we observed high levels of patient satisfaction, with 86.81% of women reporting that their care providers were caring and compassionate. This underscores the need for cautious interpretation of satisfaction measures as indicators of quality maternity care.

## Supplementary Information

Below is the link to the electronic supplementary material.

Supplementary Material 1

Supplementary Material 2

## Data Availability

The datasets analyzed during the current study, are not publicly available due to ethical considerations surrounding participant privacy but are available from the corresponding author upon reasonable request.

## Abbreviations

CCSS: Childbirth care satisfaction survey
CI: Confidence interval
COR: Crude odds ratio
CS: Caesarean section
OV: Obstetric violence
RMC: Respectful maternity care
SD: Standard deviation
SPSS: Statistical Package for the Social Sciences
WHO: World Health Organization
