# Supplementary material for: Prevalence and determinants of obstetric violence during facility-based childbirth among postnatal women in Egypt
Source: Sci Rep. 2026 Aug 17;16:25687. doi: 10.1038/s41598-026-64411-0 (PMC13482102; doi:10.1038/s41598-026-64411-0)
Supplement: Supplementary file 2 [file 41598_2026_64411_MOESM2_ESM.docx]

**Supplementary material 2**

**Questionnaire on Obstetric Violence and Associated Factors among Women in the Immediate Postpartum Period**

*This study aims to assess the prevalence of obstetric violence and its associated factors among women in Egypt. Please answer the following questions accurately. Your participation is voluntary; your identity will remain anonymous, and all information you provide will be kept confidential and used solely for research purposes.*

| 1- **Socio-demographic characteristics of the mother** | | |
| --- | --- | --- |
| Age | …………… | |
| Residence | 1. Urban area  2. Rural area | |
| Marital Status | 1. Married  2. Divorced  3. Widow | |
| Level of Education of mothers | 1. Illiterate  2. Primary  3. Secondary  4. College  5. Postgraduate studies | |
| Occupation | 1. Housewife  2. Government employee  3. Private business | |
| Social level | 1. Very low  2. Low  3. Average  4. High | |
| Number of Children | --------- | |
| Age at marriage | ----------- | |
| Educational background of the husband | 1. Illiterate  2. Primary  3. Secondary  4. College  5. Postgraduate studies | |
| Occupation of the husband | 1. Does not work  2. Government employee  3. Private business | |
| 2- **Obstetrical characteristics of the women** | | |
| Did you receive antenatal care starting from the first trimester of pregnancy? | 1. Yes  2. No | |
| Number of antenatal care visits | 1. 4 or less  2. More than 4 | |
| You received pregnancy follow-up from | 1. Physician  2. Midwife  3. Intern doctor  4. The pregnancy was not followed up | |
| Number of pregnancies | 1. 1  2. 2 or 3  3. 4 or more | |
| Number of childbirths | 1. First time  2. More than one | |
| Current place of childbirth | 1. Government hospital  2. University hospital | |
| Childbirth method | 1. Caesarean section  2. Normal vaginal using assistive devices  3. Normal vaginal with an episiotomy  4. Normal vaginal without assistance | |
| Number of people who attended the childbirth | 1. 1 to 2  2. 3 to 4  3. 5 to 8 | |
| Primary childbirth attendant | 1. Physician  2. Midwife  3. Intern doctor | |
| Sex of the primary childbirth attendant | 1. Male  2. Female | |
| Timing of childbirth | 1. During the day  2. During the night | |
| The number of days you spent in the hospital | 1. Less than 3  2. 3 or more | |
| 3- **Obstetric violence data** | | |
| **Physical violence** | | |
| Were you hit, slapped, pinched, or restrained during childbirth? | 1. Yes  2. No | |
| Was the episiotomy wound sutured without the use of anaesthesia? | 1. Yes  2. No | |
| Was your abdomen pressed hard during childbirth? | 1. Yes  2. No | |
| Was a vaginal examination performed during childbirth in a violent or unkind manner? | 1. Yes  2. No | |
| **Care without consent** | | |
| Was a caesarean section performed without your consent? | 1. Yes  2. No | |
| Was an episiotomy performed without your consent? | 1. Yes  2. No | |
| Was a hysterectomy performed without your consent? | 1. Yes  2. No | |
| Has a tubal ligation been performed without your consent? | 1. Yes  2. No | |
| Were forceps or a suction device used during the childbirth without your consent? | 1. Yes  2. No | |
| Did the childbirth take place in a public place without privacy barriers such as curtains? | 1. Yes  2. No | |
| **Non-confidential care** | | |
| Did others (other than those in charge of the childbirth) see your body during childbirth? | 1. Yes  2. No | |
| Did your childbirth team share information about you and your health with others? | 1. Yes  2. No | |
| Is your health information discussed in public places? | 1. Yes  2. No | |
| Did you experience any yelling, scolding, or blaming from the birth team? | 1. Yes  2. No | |
| **Undignified care** | | |
| Did you experience any yelling, scolding, or blaming from the childbirth team? | 1. Yes  2. No | |
| Were you treated in a harsh or humiliating manner during childbirth? | 1. Yes  2. No | |
| **Discrimination** | | |
| Have you been treated in a racist or discriminatory manner for social, educational, financial, religious, or ethnic reasons? | 1. Yes  2. No | |
| **Negligent care** | | |
| Were your requests for help ignored during childbirth? | 1. Yes  2. No | |
| Have you noticed that the obstetrician did not attend the delivery process? | 1. Yes  2. No | |
| Were you treated as a passive participant during the childbirth process? | 1. Yes  2. No | |
| Have you waited too long to get the medical care you need? | 1. Yes  2. No | |
| **Detention in the Healthcare Facility** | | |
| Were you asked to pay any money for the childbirth to take place? | 1. Yes  2. No | |
| Have you or your child been detained in the hospital until the money required for the delivery has been paid? | 1. Yes  2. No | |
| 4- **Patient Satisfaction** *Please indicate your level of agreement with the following statements using the scale below: 1 = Strongly disagree    2    3    4    5    6    7 = Strongly agree.* | | |
| 1. My experience showed that I was able to exercise adequate control over my care. | |  |
| 2. The person(s) responsible for my care were caring and compassionate. | |  |
| 3. Problems that arose during my care were managed effectively. | |  |
| 4. My needs were met within an appropriate time. | |  |
| 5. Overall, I received appropriate care. | |  |
| 6. I would choose the same type of care in a future pregnancy. | |  |
